# Toward precision psychiatry: multimodal machine learning combining neurophysiological and language features to predict symptoms severity in schizophrenia

**DOI:** 10.1192/j.eurpsy.2025.498

**Published:** 2025-08-26

**Authors:** M. Bosia, B. Scalingi, G. Agostoni, F. Pacchioni, C. Barattieri di San Pietro, P. Canal, M. F. D’Incalci, J. Sapienza, M. Bechi, F. Repaci, C. Gugielmino, R. Cavallaro, N. Dubbini, V. Bambini

**Affiliations:** 1 Vita-Salute San Raffaele University, Milano; 2 University School for Advanced Studies IUSS Pavia, Pavia; 3 IRCCS San Raffaele, Milano; 4 Miningful srls, Pisa, Italy

## Abstract

**Introduction:**

Capturing the complex and heterogeneous clinical phenotypes across Schizophrenia Spectrum Disorders (SSD) is still challenging and Artificial Intelligence is a promising tool. In the past years, machine learning (ML) models have been developed for diagnostic classification, highlighting both neuroimaging and language measures as relevant predictors (Zucchetti et al. It J of Psy (2024). Fewer studies focused on predicting symptom severity or quality of life, based on clinical variables, but with relatively low performance (Beaudoin, et al. Schizophrenia (2022); 8.1 29; Podichetty et al. Clin and Transl Sc (2021), 14(5), 1864-1874). However, to the best of our knowledge, no previous studies have applied ML regression tasks using neurophysiological features, nor combining them with language ones.

**Objectives:**

The aim is to combine neurophysiological and language features via ML to predict symptoms severity in schizophrenia.

**Methods:**

Forty-one individuals with a diagnosis of schizophrenia were enrolled and assessed for psychopathology, communicative-pragmatic abilities and underwent a 5-minute resting state EEG recordings.

A Least Absolute Shrinkage and Selection Operator (LASSO) regression was employed to identify significant EEG and language features, extracted through Natural Language Processing to predict symptoms severity. After feature selection, two LASSO models were built and compared: Model 1 (M1) included the most important EEG features, while Model 2 (M2) included both the most important EEG and language features.

**Results:**

Feature selection led to the identification of main predictors, including EEG microstates and aperiodic activity, lexical-semantic features and imageability of words.

Both models reached an acceptable performance (MSE=0.590, adj. R^2^= 0.344; MSE= 0.334, adj. R^2^ =0.582), but the Diebold-Mariano test and BIC highlighted a significant difference between M1 and M2, indicating an improved performance in predicting symptoms, when adding language features. See Fig.1 for more details

**Image 1:**

**Figure:**
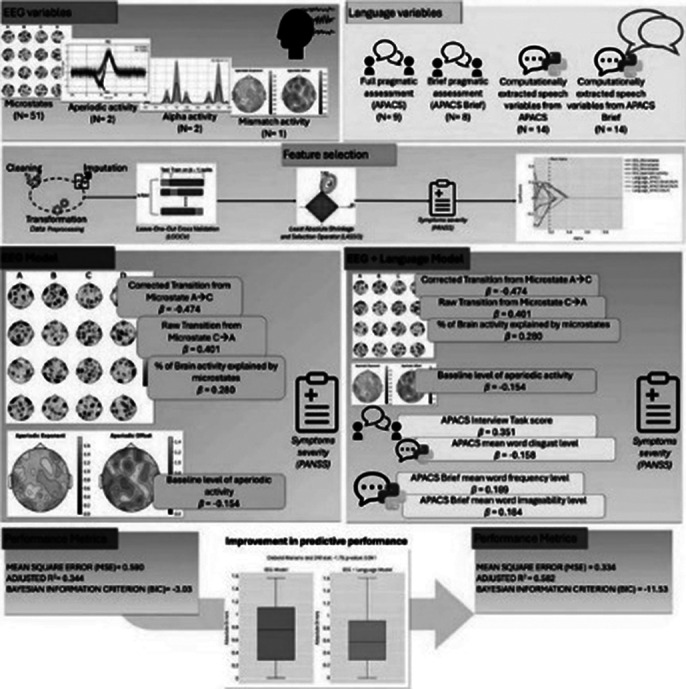

**Conclusions:**

These preliminary results identify relevant features, confirming the role of EEG and language measures as potential biomarkers for SSD. Innovatively, data also show that EEG variables alone, can reliably but only partially predict psychopathology, while the inclusion of linguistic variables further improves the model. Overall, EEG and language measures, obtained quickly through simple tasks, appear as relevant features that may discriminate clinical outcomes within SSD and implementation of ML tools mat help to guide diagnosis and refine treatments.

**Disclosure of Interest:**

None Declared

